# The Ortholog Receptor Or67d in *Drosophila Bipectinata* is able to Detect Two Different Pheromones

**DOI:** 10.1007/s10886-024-01545-3

**Published:** 2024-09-18

**Authors:** Melissa Díaz-Morales, Mohammed A. Khallaf, Regina Stieber, Ibrahim Alali, Bill S. Hansson, Markus Knaden

**Affiliations:** 1 Department of Evolutionary Neuroethology, Max Planck Institute for Chemical Ecology, Jena, Germany; 2 Max Planck Center Next Generation Insect Chemical Ecology, Jena, Germany; 3 Lankester Botanical Garden, University of Costa Rica, Cartago, Costa Rica; 4 Department of Zoology and Entomology, Faculty of Science, Assiut University, Assiut, Egypt; 5 Present Address: Department of Neuroscience, Max Delbrück Center for Molecular Medicine, 13122 Berlin, Germany

**Keywords:** *Drosophila bipectinata*, Or67d receptor, *cis*-Vaccenyl acetate, (Z)-11-eicosen-1-yl-acetate, Pheromones, Courtship behavior

## Abstract

Sex pheromones play a crucial role in species recognition and reproductive isolation. Despite being largely species-specific in drosophilids, the mechanisms underlying pheromone detection, production, and their influence on mating behavior remain poorly understood. Here, we compare the chemical profiles of *Drosophila bipectinata* and *D. melanogaster*, the mating behaviors in both species, as well as the tuning properties of Or67d receptors, which are expressed by neurons in antennal trichoid sensilla at1. Through single sensillum recordings, we demonstrate that the *D. bipectinata* Or67d-ortholog exhibits similar sensitivity to *cis*-vaccenyl acetate (*c*VA) as compared to *D.* melanogaster but in addition also responds uniquely to (Z)-11-eicosen-1-yl-acetate (Z11-20:Ac), a compound exclusively produced by *D. bipectinata* males. Through courtship behavior assays we found that, surprisingly, perfuming the flies with Z11-20:Ac did not reveal any aphrodisiacal or anti-aphrodisiacal effects in mating assays. The behavioral relevance of at1 neuron channels in *D. bipectinata* compared to *D. melanogaster* seems to be restricted to its formerly shown function as an aggregation pheromone. Moreover, the non-specific compound cVA affected copulation negatively in *D. bipectinata* and could potentially act as a premating isolation barrier. As both ligands of Or67d seem to govern different behaviors in *D. bipectinata*, additional neurons detecting at least one of those compounds might be involved. These results underscore the complexity of chemical signaling in species recognition and raise intriguing questions about the evolutionary implications of pheromone detection pathways in *Drosophila* species.

**Supplementary Information:**

The online version contains supplementary material available at 10.1007/s10886-024-01545-3.

## Introduction

Sex pheromones are communication signals used to attract conspecifics of the opposite sex for copulation (Johansson & Jones 2007; Khallaf et al. 2020). Unlike other pheromones, which can be shared among different groups of species, sex pheromones tend to be species-specific and are typically composed of a blend of molecules in a specific ratio (Wyatt 2010). This specificity, and in general, the diversification of sexual communication is one of the main evolutionary forces driving reproductive isolation and therefore speciation (Khallaf et al. 2020; Smadja & Butlin 2009).

The most studied sex pheromone in *Drosophila* is (Z)-11-octadecen-1-yl acetate (*cis*-vaccenyl acetate, *c*VA). The role of cVA in reproduction was first suggested when the compound was identified in the ejaculatory bulbs of male *D. melanogaster* flies and was shown to be transferred to females during copulation (Brieger & Butterworth 1970; Butterworth 1969). Further studies have described various functions of cVA, including reducing the sexual attractiveness of mated females (Billeter et al. 2009; Kurtovic et al. 2007; Mane et al. 1983), regulating male-male aggression (Liu et al. 2011; Wang & Anderson 2010), acting as an aphrodisiac for females (Kurtovic et al. 2007; Ronderos & Smith 2010) and promoting aggregation in the presence of food (Bartelt et al. 1985; Wertheim et al. 2002).

The detection of cVA in *Drosophila melanogaster* is mediated by the odorant receptor Or67d, which is expressed in olfactory sensory neurons (OSNs) housed in antennal trichoid sensilla 1 (at1) (Clyne et al. 1997; Ha & Smith 2006; Khallaf et al. 2021; Kurtovic et al. 2007; Wang & Anderson 2010). Other studies have shown that cVA can also activate OSNs expressing the Or65a receptor, thereby modulating different behaviors (Ejima et al. 2007; Lebreton et al. 2014; Liu et al. 2011; van der Goes van Naters & Carlson 2007). Recently, cVA was identified as a male-specific compound present in at least 34 species of *Drosophila* widely distributed across the phylogeny, making it the most common compound in the genus (Khallaf et al. 2021). Additionally, in many other *Drosophila* species OSNs housed in the at1 sensilla have been shown to respond to cVA (Khallaf et al. 2021; Prieto-Godino et al. 2020), suggesting that OR67d or its orthologs are involved.

Interestingly, some species have been found to produce cVA but not exhibit any single-sensillum responses (SSRs) from OSNs present in trichoid sensilla to it (e.g., *D. ananassae*), while other species do not produce cVA but exhibit physiological responses to it (e.g., *D. malerkotliana*) (Khallaf et al. 2021). However, the factors contributing to the discrepancy between cVA production and detection in different species within the *D. ananassae* subgroup are unknown. Herein, we approached this issue using *D. bipectinata*, which belongs to the same subgroup (Lemeunier et al. 1986), and is regarded as a model of incipient speciation (Kopp & Frank 2005). *Drosophila bipectinata* is found across a vast geographic range spanning from India and Nepal to Australia and the South Pacific islands (Bock 1971; Kopp & Frank 2005; Lemeunier et al. 1986). It has been shown that *D. bipectinata* exhibits attraction and aggregation responses to Z-11-eicosen-1-yl acetate (Z11-20:Ac) and cVA when both are combined with food odors, despite not producing cVA (Schaner et al. 1989). The neural basis for the effect of Z11-20:Ac and cVA on *D. bipectinata*'s behavior, and whether they engage shared or distinct neuronal pathways, remains unknown.

In the present study, we investigate whether the orthologous Or67d receptor of *D. bipectinata* (DbipOr67d) exhibits similar sensitivity to sex and aggregation pheromones as observed in *D. melanogaster*. Specifically, we seek to address the following questions: 1. Does the orthologous DbipOr67d receptor retain its conserved position in the OSN of at1 sensilla, akin to *D. melanogaster*? 2. Has the receptor’s tuning changed in *D. bipectinata* to detect the previously identified aggregation pheromone Z11-20:Ac (Schaner et al. 1989)? 3. Finally, does Z11-20:Ac in addition to its described function as an aggregation pheromone, also act as a sex pheromone in *D. bipectinata* flies, and by that substitute all functions that have been described for cVA in *D. melanogaster*?

## Materials and Methods

### Fly Stocks

All flies used in this study were bred on conventional cornmeal agar medium under a 12h light and 12h dark cycle at 25°C and 70% humidity. *D. bipectinata* flies were obtained from the National Drosophila Species Stock Center (NDSSC; https://www.drosophilaspecies.com/; SKU: 14,024–0381.07). Care and treatment of all flies complied with all relevant ethical regulations.

### Cloning of *D. bipectinata* Or67d Receptor and Transgenic Expression in *D. melanogaster* at1 Neuron

In order to deorphanize the *D. bipectinata Or67d* receptor, we ectopically expressed it in the so-called empty *Drosophila melanogaster* at1 neuron, i.e. a neuron that is lacking the expression of its own *D. melanogaster* OR67d receptor. To clone the full-length coding sequence of *D. bipectinata* OR67d (*Dbip*Or67d), total antennal RNA from approximately 100 *D. bipectinata* male and female flies was prepared and cDNA was synthesized using Superscript III Reverse Transcriptase kit (Thermo Fisher Scientific, Germany). The gene of *Dbip*Or67d was amplified by using PCR with the following gene-specific primers: *Dbip*Or67d-Forward: 5´ ATGTCCAAATTAAATAAAAAATTAACACCCGTTGAC 3´; *Dbip*Or67d-Reverse: 5´ CTATTTTTCCATTCCCAAGTAGGTCAGC 3´. The resulting construct of the PCR reaction (1188bp) was purified and T:A cloned into pCR^TM^8/GW/TOPO (Thermo Fisher Scientific, Germany). Sanger sequencing confirmed the sequence (Eurofins Genomics Germany GmbH and the Department of Insect Symbiosis, Max Planck Institute for Chemical Ecology, Jena).

The transfer of the construct to the destination vector pUASg.attB (kindly provided by J.Bischof, FLYORF Injection, Zürich, Switzerland) was completed as described in Gonzalez et al. (2016). Subsequently, FlyORF Injection performed the germline transformation of *D. melanogaster* with the prepared plasmid, using the PhiC31 integration system. The vector was inserted into chromosome II, fly strain attP40: 2L, 25C6 to produce the genotype + ; UAS- *Dbip*Or67/SM6a; + .

To generate the experimental fly line: + ; UAS- *Dbip*Or67d/UAS- *Dbip*Or67d; Or67d- Gal4/Or67d- Gal4, which expresses *Dbip*Or67d under regulation of Or67d in *D. melanogaster*, the following fly lines were used: + ; UAS- *Dbip*Or67/SM6a, yw;BL/Cyo; Tm2/Tm6b (obtained from Bloomington Stock Center, no. BL3704) and + ; BL/Cyo; Or67d- Gal4 no.1/Tm6b (obtained from IMP, Vienna, no. M1139).

### Thermal Desorption-Gas Chromatography-Mass Spectrometry (TD-GC–MS)

The parameters used to analyze the male-specific compounds present in *D. melanogaster* and *D. bipectinata* are described in Khallaf et al. (2021). Briefly, we used a GC–MS device (Agilent GC 7890 A fitted with an MS 5975 C inert XL MSD unit; Agilent) equipped with an HP5-MS UI column (19091S-433UI; Agilent). After desorption at 250 °C for 3 min, the volatiles were trapped at − 50 °C using liquid nitrogen for cooling. The vaporizer injector was heated gradually (12 °C/s) to 270 °C and held for 5 min. The temperature of the GC oven was held at 50 °C for 3 min, gradually increased (15 °C/min) to 250 °C and held for 3 min, and then to 280 °C (20 °C/min) and held for 30 min. For MS, the transfer line was held at 260 °C, the source at 230 °C, and quad at 150°C.

### Chemicals

All compounds used for SSR and behavior experiments, their sources, and CAS numbers are listed in Supplementary Data 1. All odors were diluted in dichloromethane (DCM) for SSR and behavioral experiments.

### Mating Assays with Perfumed Flies

Wild-type *D. bipectinata* males and females were collected after eclosion and raised individually and in groups, respectively. All mating experiments were performed under normal white light at 25 °C, 70% humidity, in a chamber (1 cm diameter × 0.5 cm depth) covered with a plastic slide to prevent the flies from escaping.

For the competition courtship experiments rival male flies were randomly marked by UV-fluorescent powder of different colors (red: UVXPBR, and blue: UVXPBY; purchased from Maxmax.com; https://maxmax.com) 24 h before the experiments. Male flies were perfumed with the compounds diluted in DCM (10 − 4 dilution (v/v)) or DCM alone. While too low concentrations might not become detected, too high concentrations of the pheromone could induce aversion. Usually assays with added pheromones are rather criticized because of the potentially too high concentrations used. However, Khallaf et al. (2020 and 2021) showed that for several species of *Drosophila* adding the pheromone in a dilution of 10^–4^ to the flies could increase the attractiveness of the male, and/or induce an antiaphrodisiac effect for the females. Therefore, we decided to follow their methodology and used the same concentration (which is added on top of their natural amount of pheromones).

Briefly, 10 μL of the solution was pipetted into a 1.5-mL glass vial and mixed with the vortex for 30 s to distribute the solution homogeneously within the vial. After evaporating the DCM under an airflow, ten flies were transferred to the vial and shaken manually for 1 min. Flies were transferred to food vials to recover for 1 h and then introduced to the courtship arenas.

Competition assays were manually observed for 1 h and copulation success was scored identifying the successful rival under UV light. Additionally, courtship behaviors were recorded for 60 min using the Noldus Media Recorder 4.0 (Noldus Information Technology, The Netherlands) coupled to a Logitech C615 webcam, at a frame rate of 30 frames per second, and a resolution of 1280 × 720 pixels in MJPG format. Each video was analyzed manually for copulation latency, which was measured as the time taken by each male until the onset of copulation, and copulation duration. Data from competition experiments represents females courted by both rival males to ensure that females or males chose between rival pairs and did not simply copulate or court with the first partner they encountered. Results from females that were only courted by one male were excluded.

In the single courtship experiments, freeze-killed virgin females were used to disentangle male sexual behaviors from female acceptance. The dead females were perfumed with (Z)-11–20:Ac diluted in DCM (10 − 4 dilution (v/v)) or DCM alone. After evaporating the DCM for ca. 1min one female and one male were placed inside the courtship arenas and recorded for 60 min. Each video was analyzed using BORIS software, version 8.24.1 (Friard & Gamba 2016) to obtain the courtship index, which was calculated as the percentage of time that the male spends courting the female during 20 min of observation, after giving them 10 min for acclimation in the chamber.

### Electrophysiology

SSR were performed following the protocol described by Olsson and Hansson (2013). Adult male and female flies (6–10 days old) were immobilized in a 100 µl plastic pipette tip with the wide end closed with dental wax (Erkodent, Germany) and the narrow end cut to allow only half of the head with the antennae to protrude. The third antennal segment was placed in a stable position on a glass coverslip in a ventrolateral position, and the funiculus of the antenna was fixed with a sharpened glass capillary (placed between the second and third antennal segment).

Trichoid sensilla were identified based on their morphology under a microscope (Olympus BX51WI) at 100 × magnification. The targeted at1 sensilla were identified based on their central location in the antenna and its spontaneous activities (single neuron), which are known in *D. melanogaster* (Lin & Potter 2015; Miller & Carlson 2010). Stimulus cartridges were prepared by pipetting 10 μl of the diluted odor onto a 1 cm ⌀ piece of filter paper (Rotilabo type 601A, Carl Roth, Germany), placed inside a Pasteur pipette using as lid a 1 ml plastic pipette clogged with wax to prevent evaporation. We used dilutions at a concentration of 10^–3^ (v/v), using dichloromethane (DCM) (Sigma-Aldrich, Germany) as diluent as described in Khallaf et al. (2021) for the same compounds. We tested a wide array of chemicals including the cuticular hydrocarbons (CHCs) characterized for *D. melanogaster* and *D. bipectinata*, compounds that are structurally similar to those (i.e., Z-9–16:Ac, Myristyl acetate, Z-11–16:Ac, Z-9-Tricosene, Z-9–18:Ac), plus additional CHCs described from other *Drosophila* species. Dose–response curves were prepared for those compounds that elicited positive responses in the wild-type *D. melanogaster* and *D. bipectinata* flies. We tested dilutions at 10^–1^, 10^–2^, 10^–3^, 10^–4^, and 10^–5^ (v/v) concentrations, using DCM as diluent.

The fixed fly was continuously flushed by clean air through a plastic tube of 0.4 cm diameter delivering a 1.0 lpm (l/min) flux of charcoal-filtered and humidified air located ca. 5 cm from the preparation. The antenna of the fly was stimulated by a 0.5 s air pulse (0.5 lpm) through the stimulation cartridge located ca. 10 mm from the antenna (Ng et al. 2017). The entire odorant panel was tested on one to three sensilla per fly. The extracellular signals originating from the OSNs were measured by inserting a tungsten wire electrode in the base of a sensillum and a reference electrode into the eye. Signals were amplified (Syntech Universal AC/DC Probe; Syntech), sampled (9,600 samples/s), and filtered (300–3000 Hz with 50/60 Hz suppression) via USB-IDAC connection to a computer (Syntech).

Action potentials were extracted using AutoSpike software, version 3.10 (Syntech). Neuron activities were recorded for 5 s, starting 2 s before a stimulation period of 0.5 s. Neural responses were calculated as the increase (or decrease) in the action potential frequency (spikes/s) relative to the pre-stimulus frequency by manually counting the number of spikes one second before and one second after the beginning of the stimulus.

### Statistical Analysis

The normality test was first assessed on datasets using a Shapiro test. Statistical analyses (see the corresponding legends of each figure) and preliminary figures were conducted using R version 4.3.0 (R Core Team 2024). Figures were then processed with Adobe Illustrator CS5.

## Results

### Chemical Profiles of Males and Females of *D. bipectinata*

**Fig. 1:**
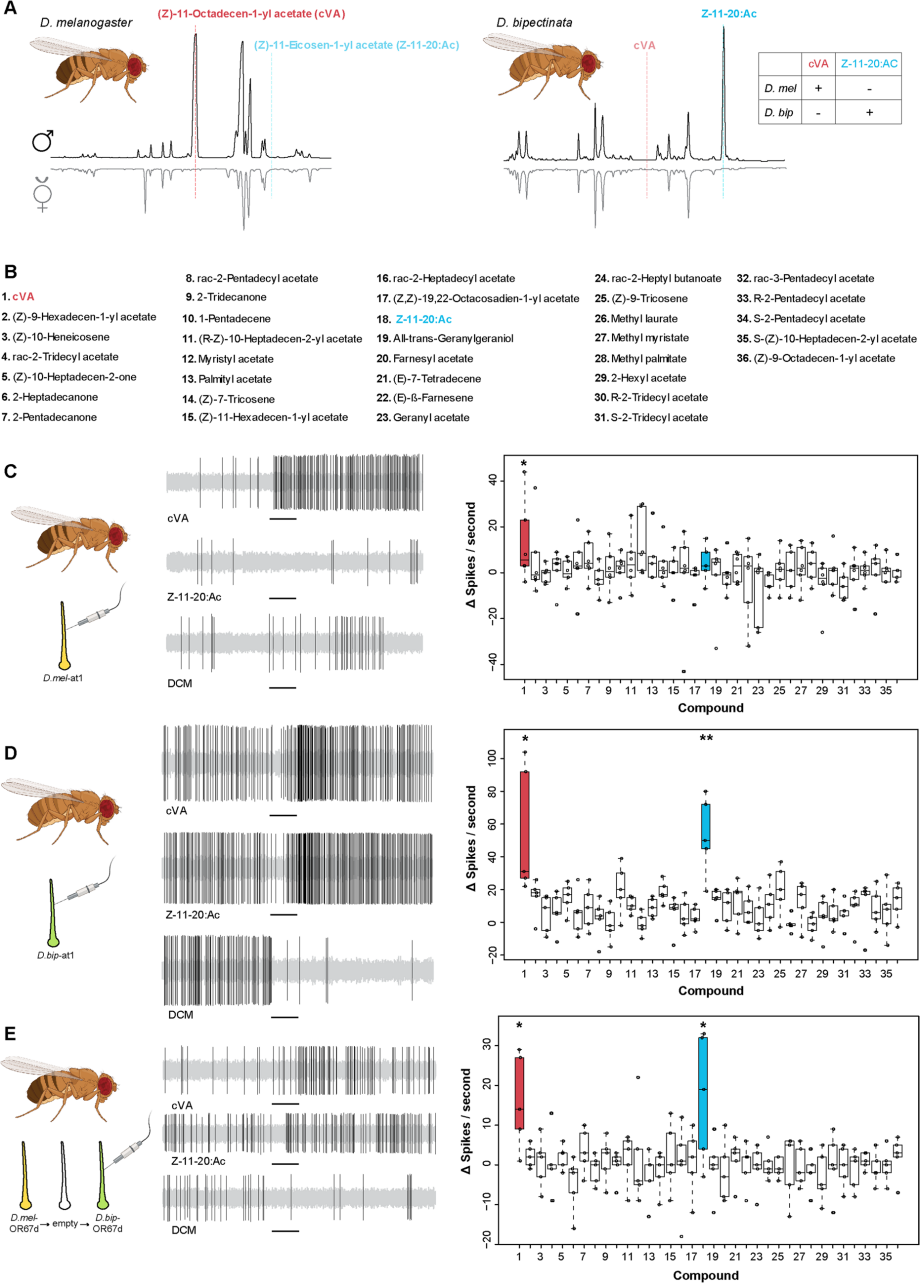
SSR responses from the OSN present in at1 sensilla. **A.** Representative gas chromatograms of male and female flies obtained by solvent-free thermal desorption–gas chromatography–mass spectrometry (TD-GC–MS). *Drosophila melanogaster* (left); red line, presence of cVA in male flies; light-blue line, absence of Z11-20:Ac. *Drosophila bipectinata* (right); light-red line, absence of cVA; blue line, presence of Z11-20:Ac. Summary table for the presence and absence of the two male-specific compounds of *D. melanogaster* and *D. bipectinata*. **B.** List of the tested male-specific compounds. **C.** OSNs in *Drosophila melanogaster* CS at1 sensilla respond to cVA but not to Z11-20:Ac. **D.** OSNs in *Drosophila bipectinata* respond to cVA and Z11-20:Ac. **E.** *Drosophila melanogaster* OSNs ectopically expressing D.bipOr67d respond to cVA and Z11-20:Ac. For all tested flies the solvent (DCM) was used as control. Black lines under electrophysiological traces represent time and duration of stimulus (0.5 s). Red box plots, responses to cVA; blue box plots, responses to Z11-20:Ac; white box plots, responses to other male specific compounds described in *Drosophila* species (Khallaf et al. 2021). Asterisks depict statistical significances (*p < 0.05; **p < 0.01; n = 5–6; One sample *t* test)

To compare candidate pheromones potentially mediating sexual behaviors in *D. melanogaster* and *D. bipectinata*, we analyzed the chemical profiles of males and virgin females of each species. Our analysis revealed that *D. bipectinata* males possess two compounds, (Z)-11-eicosen-1-yl acetate (Z11-20:Ac) and palmityl acetate, which are not found in *D. bipectinata* females or in *D. melanogaster* (Fig. 1a; data not shown for palmityl acetate). Additionally, *D. bipectinata* males lack cis-vaccenyl acetate (cVA), the male-specific compound produced by *D. melanogaster* male flies. These results are consistent with the previous chemical analyses reported by Khallaf et al. (2021).

### Responses of the Orthologous Or67d to Male-Specific Compounds

To test for differences in the electrophysiological responses of the orthologous Or67d receptor in *D. melanogaster* and *D. bipectinata* we performed SSR from OSNs present in at1 sensilla of both species. We tested a set of 36 male-specific compounds previously identified from ca. 80 different species of drosophilid flies (Fig. 1b) (Khallaf et al. 2021). We found that *D. melanogaster* CS flies exhibited significant responses only to cVA (Fig. 1c), their sex pheromone to which Or67d has been reported to be tuned (Clyne et al. 1997). Interestingly, OSNs housed in at1 sensilla of wild-type *D. bipectinata* flies responded similarly to cVA but also, and uniquely, to Z11-20:Ac (Fig. 1d), one of the two male-specific compounds identified in this species (Khallaf et al. 2021). We obtained the dose–response curves to the compounds cVA and Z11-20:Ac in both species (Suppl. Figure 1). We observed that *D. bipectinata* flies respond with similar intensity to both compounds, reaching saturation at 10^–3^ (v/v), while the cVA responses in *D. melanogaster* are at the same time similar to those of *D. bipectinata* (Suppl. Figure 1). Our findings, demonstrating consistent response kinetics and cVA detection by the at1 sensilla OSNs in both species, indicate the conservation of the Or67d orthologous receptor’s position in the OSN present in at1 sensilla. Furthermore, when recording from *D. melanogaster* at1 OSNs ectopically expressing the orthologous DbipOR67d, we found positive responses to the same compounds as observed in the wild-type *D. bipectinata* (Fig. 1e). This confirms that, while the DbipOr67d receptor maintains its position in at1, its tuning properties have evolved to additionally detect another compound (Z11-20:Ac), produced by males of *D. bipectinata*.

### Courtship Behavior

**Fig. 2:**
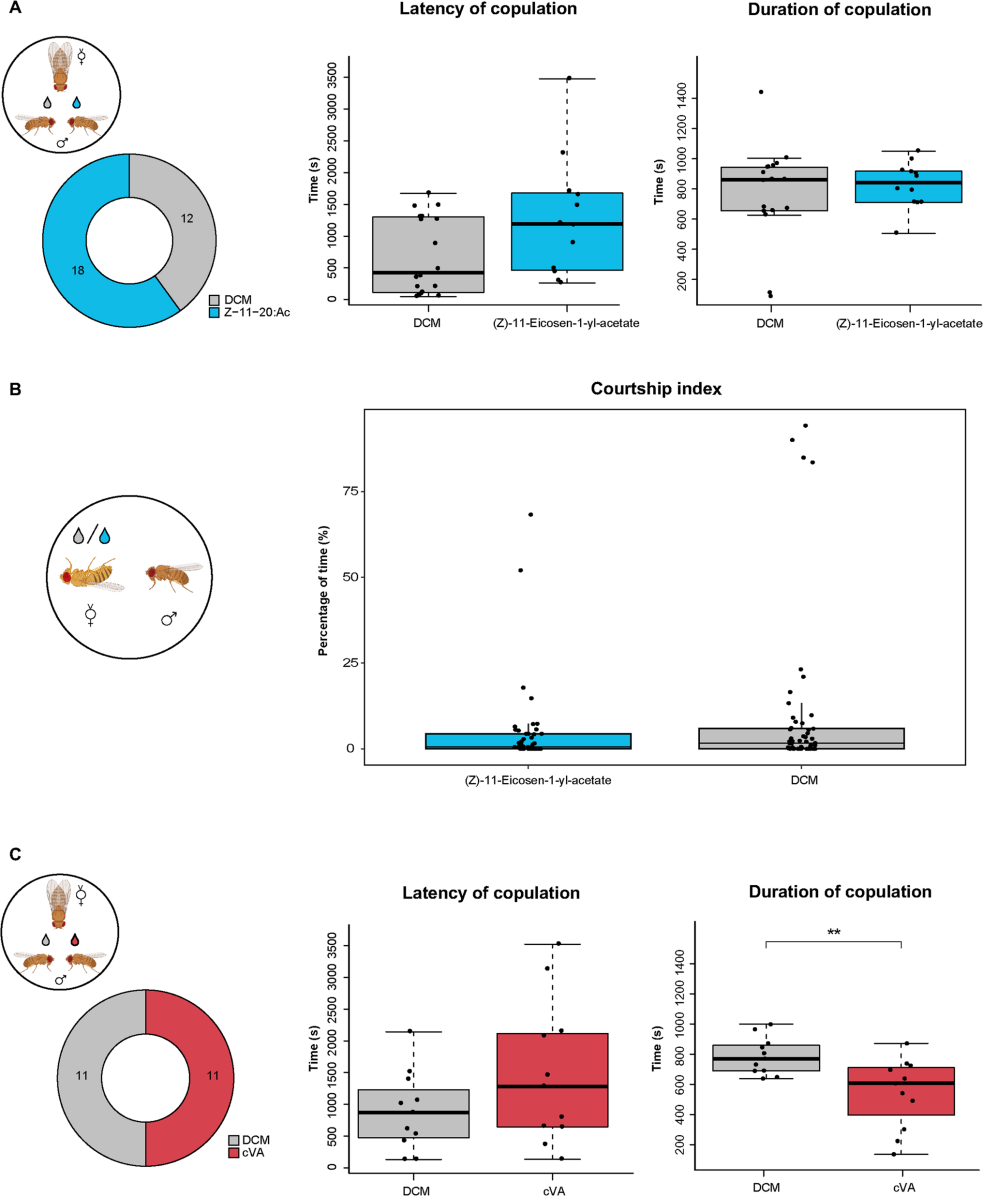
Courtship behavior of *D. bipectinata* flies. **A.** Assay to test for attractiveness of males perfumed with Z11-20:Ac or the solvent (DCM) towards females. Doughnut plot, female choice (blue, pheromone-perfumed male chosen; grey, control male chosen; *p* > 0.05; n = 30; *χ*^2^ test). Boxplots, mating latency (left) and duration of copulation (right) with pheromone-perfumed (blue) or control (grey) males (*p* > 0.05; n = 12–18; Wilcoxon rank sum test). **B.** Assay to test for courtship inhibition towards females perfumed with Z11-20:Ac or the solvent (DCM) (*p* > 0.05; n = 48–52; Wilcoxon rank sum test). **C.** Assay to test for attractiveness of males perfumed with cVA or the solvent (DCM). Doughnut plot, female choice (red, pheromone-perfumed male chosen; grey, control male chosen; *p* > 0.05; n = 22; *χ*.^2^ test). Left box plot, latency of copulation (*p* > 0.05; n = 11; Two sample *t* test); right box plot, duration of copulation (**p < 0.01; n = 11; Two sample *t* test)

To test if Z11-20:Ac makes *D. bipectinata* males more attractive to females, similar to the effect of cVA in *D. melanogaster*, we conducted competition courtship experiments. Male flies were perfumed with either the compound or a solvent. Perfuming the flies with a given compound could tell, whether the increase of this compound affects a male’s attractiveness as shown for some species and their respective male-specific compounds (Khallaf et al. 2021). We found that adding Z11-20: Ac did not make males more attractive to females, as there was no significant difference in the proportion of females that preferred to mate with males perfumed with Z11-20:Ac compared to those perfumed with the solvent (Fig. 2a). Additionally, there were no differences in the latency or duration of the copulation (Fig. 2a).

Since Z11-20:Ac has been reported to be transferred to females during mating (Schaner et al. 1989; but see Khallaf et al. 2021), we investigated whether this compound, similar to the role of cVA in *D. melanogaster*, might reduce the attractiveness of the females to other males. In single courtship assays, we tested this by perfuming freeze-killed females and measuring the males’ courtship duration. Our results showed that male flies courted perfumed females similarly long, regardless of whether the females were perfumed with Z11-20:Ac or the solvent (Fig. 2b). Therefore, we conclude that, unlike cVA in *D. melanogaster*, Z11-20:Ac in *D. bipectinata* does not inhibit male courtship behavior towards mated females.

Although *D. bipectinata* does not produce any cVA, the response to this compound by DbipOr67d is conserved (Fig. 1c and d). We, thus, asked whether artificially applying cVA on *D. bipectinata* males might still increase their attractiveness toward females. As expected from the negative results from Z11-20:Ac (Fig. 2a), which is detected by the same receptor (Fig. 1c and d), cVA did not increase the males’ attractiveness (Fig. 2c). In contrast to our expectations, males with cVA took even tendentially longer in convincing females to mate and exhibited a significantly shorter mating duration as compared to control males (Fig. 2c). Thus, while Z11-20:Ac alone did not influence *D. bipectinata* courtship behavior, the non-specific compound cVA affected copulation success in this species negatively. We conclude that in *D. bipectinata* Z11-20:Ac, which like cVA in *D. melanogaster* is detected by Or67d, fulfills the function of cVA as an aggregation pheromone (Schaner et al. 1989), but neither governs male attraction nor courtship inhibition towards mated flies.

## Discussion

Our study investigated the role of the orthologous DbipOr67d receptor in detecting male-specific compounds. We found that this receptor retains its position in the OSN present in at1 sensilla and responds to Z11-20:Ac and cVA in *D. bipectinata* flies and in transgenic *D. melanogaster* expressing DbipOr67d in at1 OSNs. All pheromone-detecting receptors identified for *Drosophila* are expressed in olfactory sensory neurons housed in trichoid sensilla (Fishilevich & Vosshall 2005; Lin & Potter 2015; van der Goes van Naters & Carlson 2007). Given that Z11-20:Ac and cVA have very similar structures (with cVA being just 2-carbons shorter) it is not entirely unexpected that DbipOr67d can be activated by both molecules. It is however interesting that of both similar compounds only cVA activates the *D. melanogaster* Or67d receptor. While in Khallaf et al. 2021 two additional compounds ((Z)-7 Tricosene and (Z)-11 Hexadecen-1-yl acetate) activated the OR67d-expressing neuron of *D. melanogaster*, maybe due to the variability of data, the observed responses in our study to those compounds were not significant.

The activation of the Or67d receptor in *D. melanogaster* is mediated by LUSH, a pheromone-binding protein (PBP) that is secreted into the lymph of all trichoid sensilla (Billeter & Levine 2015; Rihani et al. 2021; Xu et al. 2005). Although we did not investigate the role of PBPs in *D. bipectinata*, the binding of Z11-20:Ac and cVA to the DbipOr67d receptor could also be mediated by these proteins, as is the case with other pheromones (Campanacci et al. 2001; Rihani et al. 2021). However, as we observed a specific response pattern of the *D. bipectinata* OR67d receptor even when being expressed in the *D. melanogaster* empty neuron with all the *D. melanogaster* PBPs, the difference should be caused by the changed OR67d sequence. Future investigations that include structural analyses of Or67d orthologs and their corresponding ligands of more species might shed light on the coevolution of pheromones and the corresponding receptors.

Since *D. bipectinata* flies have been shown to aggregate towards Z11-20:Ac and cVA (Schaner et al. 1989), we sought to test if these compounds could also act as sex pheromones, similar to how cVA functions in *D. melanogaster*. Specifically, we focused on Z11-20:Ac, which is produced by *D. bipectinata* (Khallaf et al. 2021; Schaner et al. 1989). Our bioassays suggest that neither Z11-20:Ac nor cVA increased the attractiveness of males, nor did Z11-20:Ac exhibit any anti-aphrodisiacal effect when applied on females.

This could have several explanations. It is possible that Z11-20:Ac mediates aggregation but not copulation in *D. bipectinata*. Khallaf et al. (2021) identified palmityl acetate as another male-specific compound produced by the species, but the receptor tuned to this compound and its potential role in the mating behavior of the species still remain to be explored. Alternatively, Z11-20:Ac could act synergistically with food odors to mediate mating, as also the cVA-governed attraction of male *D. melanogaster* flies towards females is synergistically increased in the presence of food odors (Das et al. 2017). Furthermore, it is known that the behaviors or physiological responses elicited by pheromones can be modulated by many factors including context, time of the day, hormonal state, and previous experiences (Wyatt 2017). Unfortunately, the lack of information about the natural history of *D. bipectinata* impeded further investigations in this direction.

*Drosophila* flies from the *D. mojavensis* species complex – like *D. bipectinata* – do not produce cVA; nevertheless, it has been shown that the OSNs in their trichoid sensilla also exhibit responses to cVA (Khallaf et al. 2021). Furthermore, Khallaf et al. (2021) demonstrated that perfuming male flies of this species complex with cVA makes them less attractive to virgin females. This observation indicates that non-specific compounds could still be detected and influence mating behavior and potentially reinforce sexual isolation. Our results showed that, although cVA does not prevent *D. bipectinata* flies from mating, it reduces the duration of copulation. The mechanism behind this effect is unclear, but we can speculate that the proximity during copulation may allow the female to detect the foreign pheromone (cVA in this case) prompting her to interrupt the copulation as a strategy to avoid being inseminated by males of the wrong species. As cVA and Z11-20:Ac both activate Or67d, the difference in behavior probably is mediated by the detection of cVA through another olfactory receptor. From *D. melanogaster* it is known, that cVA becomes not only detected by Or67d, but also by neurons expressing the Or65a receptor (Ejima et al. 2007; Lebreton et al. 2014; Liu et al. 2011; van der Goes van Naters & Carlson 2007). Interestingly the neurons expressing Or67d and Or65a – although detecting cVA – govern different behaviors. In Or67d neurons, cVA elicits courtship and male-male aggression, while in Or65a neurons, long-lasting exposure to cVA suppresses courtship and male-male aggression (Lebreton et al. 2014; Liu et al. 2011). Future studies might reveal, whether any additional receptors are involved in the different behaviors elicited by cVA and Z11-20:Ac.

Studies have shown that species from the *D. bipectinata* species complex can hybridize and that the divergence of this species complex is relatively recent, occurring between 283,000 and 385,000 years ago (Bock 1978; Kopp & Barmina 2005). Additionally, morphological differences among females are almost absent, with the species being distinguishable primarily by the morphology of the sex combs and abdominal pigmentation of the males (Singh & Singh 2001). Some of these species are sympatric; for example, both *D. bipectinata* and *D. malerkotliana* share much of their geographical distribution in Southeast Asia (Singh & Singh 2001), and they both have Z11-20:Ac as part of their cuticular hydrocarbon bouquet (Khallaf et al. 2021; Schaner et al. 1989).

How *D. bipectinata* recognizes its conspecifics and avoids hybridization in nature is not yet understood. Our study, for the first time, shows that the tuning of an ortholog of the most studied pheromone receptor in *Drosophila* has changed to detect a new compound. This opens new questions for investigating the evolution of the receptor across the phylogeny of the genus, shedding light on how it has adapted to detect novel compounds and the ecological roles they play.

## Supplementary Information

Below is the link to the electronic supplementary material.

Supplementary file1 (PDF 138 KB)

## Data Availability

No datasets were generated or analysed during the current study.
