# Supplementary material for: The Ortholog Receptor Or67d in Drosophila Bipectinata is able to Detect Two Different Pheromones
Source: J Chem Ecol. 2024 Sep 18;50(11):610–9. doi: 10.1007/s10886-024-01545-3 (PMC11543753; doi:10.1007/s10886-024-01545-3)
Supplement: Supplementary file 1 — Supplementary file1 (PDF 138 KB) [file 10886_2024_1545_MOESM1_ESM.pdf]

Supplementary Table 1. Names, chemical classes, source, and vapor pressure at 25 °C of the compounds used in the SSR experiments.

Compound Name	Chemical class	Vendor/Supplier	CAS Number	Vapor pressu (mmHg @ 25
(*Z*)-11-Octadecen-1-yl acetate	Ester	AK Scientific, Inc.	6186-98-7	0.000438
(*Z*)-9-Hexadecen-1-yl acetate	Ester	synthesized by Jerrit Weißflog		0.00448
(*Z*)-10-Heneicosene	Alkene	synthesized by Jerrit Weißflog		0.0000873
rac-2-Tridecyl acetate	Ester	synthesized by Jerrit Weißflog		0.004
(*Z*)-10-Heptadecen-2-one	Ketone	synthesized by Jerrit Weißflog		NA
2-Heptadecanone	Ketone	synthesized by Jerrit Weißflog		0.001
2-Pentadecanone	Ketone	Sigma-Aldrich	2345-28-0	0.003
rac-2-Pentadecyl acetate	Ester	synthesized by Jerrit Weißflog		NA
2-Tridecanone	Ketone	Sigma-Aldrich	593-08-8	0.016
1-Pentadecene	Alkene	Sigma-Aldrich	13360-61-7	0.00454
R-(*Z*)-10-Heptadecen-2-yl acetate	Ester	synthesized by Jerrit Weißflog		0.003
Myristyl acetate	Ester	AKos Consulting & Solutions	638-59-5	0.001
Palmityl acetate	Ester	AKos Consulting & Solutions	629-70-9	0.00026
(*Z*)-7-Tricosene	Alkene	AKos Consulting & Solutions	52078-57-6	0.0047
(*Z*)-11-Hexadecen-1-yl acetate	Ester	synthesized by Jerrit Weißflog		0.00367
rac-2-Heptadecyl acetate	Ester	synthesized by Jerrit Weißflog		0.003
(*Z*,*Z*)-19,22-Octacosadien-1-yl acetate	Ester	synthesized by Jerrit Weißflog		0.0000825
(*Z*)-11-Eicosen-1-yl acetate	Ester	synthesized by Jerrit Weißflog		0.0001
All-trans-Geranylgeraniol	Alcohol	Sigma-Aldrich	24034-73-9	NA
Farnesyl acetate	Ester	synthesized by Jerrit Weißflog		0.000054
(*E*)-7-Tetradecene	Alkene	Alfa Chemistry	41446-63-3	0.0322
(*E*)-ß-Farnesene	Alkene	Sigma-Aldrich	18794-84-8	0.01
Geranyl acetate	Ester	Sigma-Aldrich	105-87-3	0.07
rac-2-Heptyl butanoate	Ester	Alfa Chemistry	39026-94-3	0.09
(*Z*)-9-Tricosene	Alkene	Sigma-Aldrich	27519-02-4	0.0000353
Methyl laurate	Ester	Sigma-Aldrich	111-82-0	0.0041
Methyl myristate	Ester	Sigma-Aldrich	124-10-7	0.00049

Methyl palmitate	Ester	Sigma-Aldrich	112-39-0	0.000149
2-Hexyl acetate	Ester	synthesized by Jerrit Weißflog		NA
R-2-Tridecyl acetate	Ester	synthesized by Jerrit Weißflog		0.004
S-2-Tridecyl acetate	Ester	synthesized by Jerrit Weißflog		0.004
rac-3-Pentadecyl acetate	Ester	synthesized by Jerrit Weißflog		NA
R-2-Pentadecyl acetate	Ester	synthesized by Jerrit Weißflog		NA
S-2-Pentadecyl acetate	Ester	synthesized by Jerrit Weißflog		NA
S-(*Z*)-10-Heptadecen-2-yl acetate	Ester	synthesized by Jerrit Weißflog		0.003
(*Z*)-9-Octadecen-1-yl acetate	Ester	synthesized by Jerrit Weißflog		0.000001
(*Z*)-7-Pentacosene	Alkene	Biomol GmbH	63623-49-4	NA

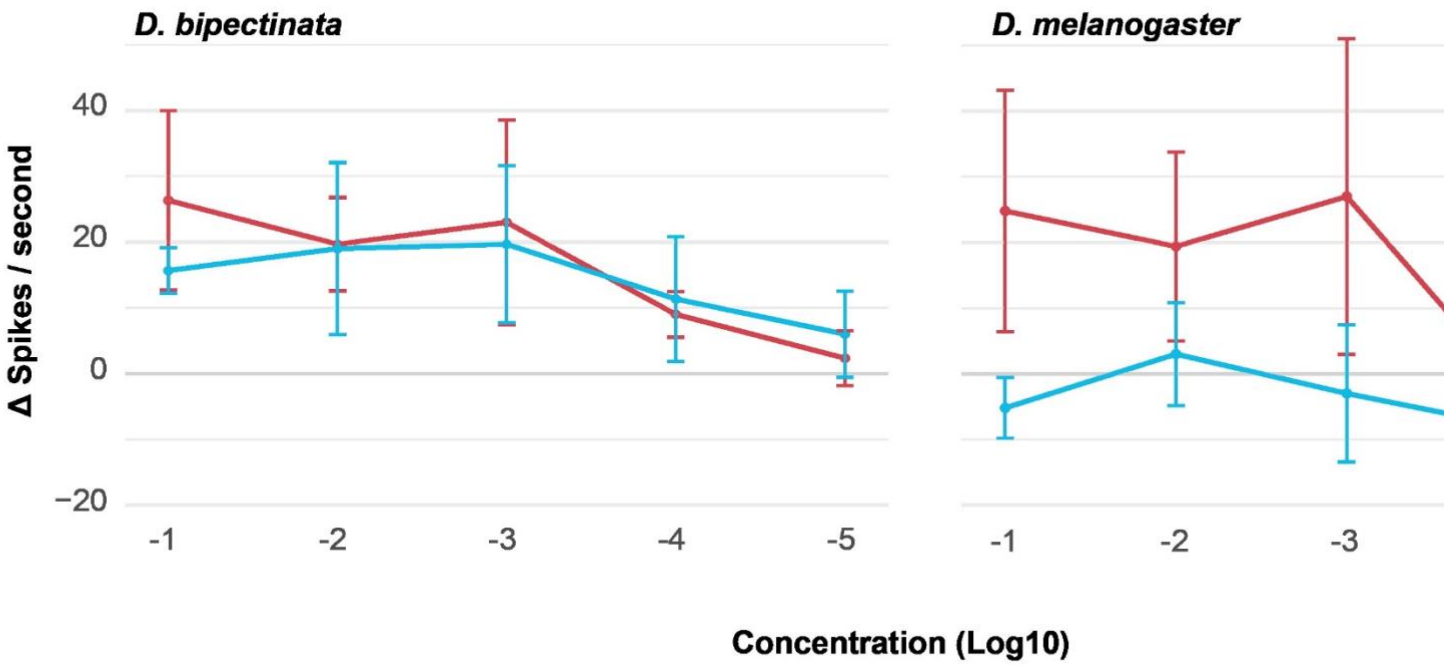

Supplementary Figure 1. SSR responses from the OSN present in at1 sensilla to 10^{-1}, 10^{-2}, 10^{-3}, 10^{-4}, and 10^{-5} (v/v) concentrations of cVA and Z11-20:Ac. ***Drosophila bipectinata*** (left); red line, responses to cVA; blue line, responses to Z11-20:Ac; n = 3. ***Drosophila melanogaster*** (right); red line, responses to cVA; blue line, responses to Z11-20:Ac; n = 5. For all tested flies the solvent (DCM) was used as control.
